# Effect of dentin surface pretreatment with four conditioning agents on micro-shear bond strength of a self-adhesive cement

**DOI:** 10.4317/jced.59438

**Published:** 2022-05-01

**Authors:** Wilson Choque-Apaza, Marco Sánchez-Tito

**Affiliations:** 1 Private practice, Tacna, Peru; 2 Facultad de Ciencias de la Salud, Universidad Privada de Tacna, Tacna, Peru

## Abstract

**Background:**

To evaluate the effect of dentin surface pretreatment with four conditioning agents on the micro-shear strength of a self-adhesive cement.

**Material and Methods:**

32 specimens of 6 mm high x 4 mm wide with dentin exposure were obtained and divided into four groups of NaOCI, CHX, EDTA and AgNPs (n = 8). 2 mL of each treatment agent was applied to the dentin for 60 seconds. Composite resin cylinders measuring 3 mm x 2 mm were cemented with RelyX U200 self-adhesive cement. Micro-shear testing was performed after 24 hours and one week (n = 4) with a 1 mm/min; failure values were recorded in MPa. The failure pattern was evaluated with a stereomicroscope at 20x. The Kruskal-Wallis test was used to evaluate differences between groups. The Mann-Whitney U test was used to evaluate between evaluation times. The significance level used was *p*<0.05.

**Results:**

At 24 hours after cementation, the highest value of micro-shear bond strength was observed for chlorhexidine (15.951 MPa), this value were similar for NaOCl 4% and EDTA, however significant differences were observed when compared with AgNPs (*p*<0.05). When compared the values at 24 hours and after one week, no differences were observed (*p*>0.05). The most frequent failure pattern was mixed, followed by adhesive failure.

**Conclusions:**

Pretreatment of dentin with sodium hypochlorite, CHX and EDTA positively affected the 24-hour bonding capacity of RelyX U200 self-adhesive resin cement, although it decreased after one week.

** Key words:**Dental cements, disinfectants, calcium chelators, nanoparticles.

## Introduction

Self-adhesive resin cements are a clinically attractive option for restorative procedures in dentistry (1,2). The self-adhesive property of these cements depends on their main components’ physical and chemical properties (3).

Unlike conventional resin cements that contain an organic matrix and high molecular weight monomers, such as bisphenol-A-diglycidyl methacrylate (Bis-GMA), urethane dimethacrylate (UDMA), low molecular weight monomers, such as triethylene glycol dimethacrylate (TEGDMA) and hydroxyl ethyl methacrylate (HEMA. Self-adhesive cements mainly contain methacrylate monomers with carboxylic acid groups, such as 4-methacryloyloxyethyl trimellitic anhydride (4-META), or phosphoric acid groups, such as 10-methacryloyloxydodecyl dihydrogen phosphate (MDP) (4).

On the other hand, the moisture content of dentin can affect the bonding response of restorations to the substrate. A very wet dentin surface can cause emulsification and produce holes in the primer; conversely, a dry dentin surface can cause collagen fibers to collapse, reduce resin penetration, and create pores under the restorative material (5,6). Thus, several approaches have been proposed for self-adhesive restorations and different types of adhesive systems that could promote and improve bond strength through techniques involving the pretreatment of dentin surfaces (7-9).

Sodium hypochlorite (NaOCI) is the most commonly used irrigant in the chemical-mechanical preparation of root canals (10). Its oxidizing properties can create an oxygen-rich layer on the dentin wall that inhibits resin polymerization and increases microleakage, resulting in reduced bond strength of various adhesive systems to root canals. However, several studies have proposed its use to improve adhesion. Cecchin *et al*. observed that the use of NaOCl did not generate a decrease in micro-shear strength when the XeNO III self-etching adhesive system was used on dentin (11). On the other hand, chlorhexidine gluconate (CHX) has been recommended as an alternative irrigant with antimicrobial action, low toxicity, and the ability to remain active at the site of action (12). The use of CHX as an agent prior to adhesive procedures does not interfere with immediate bond durability, and significantly, higher bond strengths have been observed after only 6 to 12 months by inhibiting collagen-degrading metalloproteinase (MMP) enzymes (13,14).

It is known that the smear layer can be removed by different procedures, such as the use of chelating agents or the use of acids, such as polyacrylic and phosphoric acids. Total or partial removal of the smear layer occurs depending on the time and concentration of these substances (15). Faria-e-Silva *et al*. evaluated the effect of intraarticular dentin treatment with EDTA on the retention of fibreglass posts cemented with self-adhesive resin cement concluded that BisCem cement was the only material in which pretreatment of dentin with EDTA improved the bond strength to root dentin (6.0 MPa) compared to the control group (4.4 MPa), polyacrylic acid (3.6 MPa) and NaOCI (5.3MPa) (16).

The use of silver nanoparticles (AgNPs) has been incorporated into various adhesive materials and has been shown to possess an antibacterial effect on the bacterial biofilm present on the restoration surface (17). Jowkar *et al*. evaluated the effect of pretreatment of dentin with silver nanoparticles (AgNPs) and a chlorhexidine (CHX) on the micro-shear bond strength of different dentin adhesives, they concluded that the application of AgNPs was associated with higher micro-shear strength than that observed in the CHX and control groups for the Clearfil SE Bond (SEB) adhesive system after 24 hours (*p* < 0.05). They observed that the µSBS values of the 6-month samples were significantly lower than those obtained from the 24-hour samples (18).

The objective of this study was to evaluate the effect of dentin treatment with sodium hypochlorite, chlorhexidine, EDTA and AgNPs solution on the micro-shear bond strength of a self-adhesive resin cement.

Material and methods

-Study design and sample size calculation

An experimental, *in vitro*, cross-sectional, analytical and prospective study was carried out. To calculate the sample size, the G*power program was used with an effect size of 0.65, and alpha error of 0.05 and a power of 0.8, calculating a total of 32 samples. The study was approved by the Research Ethics Committee of the Faculty of Health Sciences of the Universidad Privada de Tacna, under protocol No. 002-2021-UPT/FACSA. Third molars collected from private dental offices were used for this study, where the patients signed an informed consent form. The inclusion criteria were: teeth without caries or restorations, extracted for orthodontic or prophylactic reasons, with a post-extraction time of no more than three months.

-Sample preparation

The teeth were immersed in 2% glutaraldehyde (Glutfar plus HLD) to remove soft tissue debris. Subsequently, the samples were cleaned with brushes and prophylactic paste (Shine, Maquira, Paraná, Brazil) using a low-speed piece (Sigma CX235-1st, Foshan, China). Finally, they were washed and stored in 2% glutaraldehyde until use.

The crowns of the teeth were sectioned longitudinally at the level of the main fossa in the mesiodistal direction, and another cut in the vestibular-palatal direction with a diamond disk (Komet K6974 disk, Lemgo, Germany) at low speed with copious water cooling. The teeth were decoronated at the level of the amelo-cemental junction. Subsequently, each specimen was cut mesiodistally with a diamond disc under cooling to 2.5 mm for dentin exposure. The final size of the specimens was 6 mm high x 4 mm wide. Four sections were obtained for each tooth, and a total of 32 specimens were randomly divided into four groups (n = 8). Subsequently, the specimens of each group were divided into two subgroups (n = 4) to evaluate the resistance to micro-shear bond strength in two time periods (T1 = 24 hours, T2 = 1 week).

The tooth samples were polished sequentially with water sandpaper # 400, #600, #800, #1000 (ABRALIT, Lima, Peru) to standardize the surfaces with 8-shaped movements for 10 seconds (17). The samples were individually embedded in self-curing acrylic, contained in 3/4 PVC test tubes of 19 mm diameter and 15 mm height. The samples were stored in distilled water until use.

Polystyrene tubes with an internal diameter of 2 mm and a length of 3 mm (Tygon Medical Tubing Formulations 54-HL, Saint Gobain Performance Plastics; Akron, Ohio) were used as molds to make the resin cylinders. The tubes were filled with composite resin (FiltekZ250TM, 3M ESPE, St, Paul, USA) and light-cured with an LED lamp (LED. H, WOODPECKER, Guilin, China) with an intensity of 1000Mw/cm² for 30 seconds and stored in distilled water.

Before cementation, the dentin surface was subjected to four conditioning agents: 4% sodium hypochlorite (Clorox Peru, Callao, Peru), EDTA (biodynamic, Ibipora, Brazil), 2% chlorhexidine (Maquira, Maringá, Brazil) and AgNPs (U.S. Research Nanomaterials, Inc, Houston, TX USA). Details of the products used in this study are shown in Table 1. In the case of AgNPs, 200 mL of a 23 ppm solution was prepared by diluting 4.6 mg of AgNPs in 200 mL of distilled water. Finally, 2 ml of each agent was directly applied with a sterile syringe to the dentin (17). The solutions were applied for 60 seconds in all cases. For the NaOCI 4% and EDTA group, the specimens were washed for 20 seconds with distilled water. For the CHX 2% and AgNPs groups, the specimens were dried for 20 seconds.

**Table 1:**
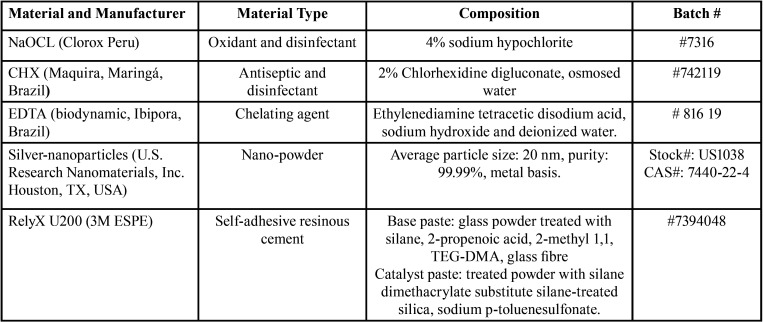
Materials used in the study.

-Bonding procedure 

RelyXTM U200 resin cement was used in a 1:1 base paste/catalyst paste ratio; prepared according to the manufacturer’s instructions. The self-adhesive cement was applied on the dentin surface previously treated with the agents, and an oil-free air jet was applied for 10 seconds to spread the cement, and the composite resin cylinders were placed. A constant force of 50 grf was applied with a dynamometer for 30 seconds to standardize the thickness of the cement, and light-cured for 40 seconds; finally the samples were stored in distilled water at 37°C (19).

After 24 hours of storage, the samples were placed in a universal testing machine (OM 150; Odeme dental research; S.C., Brazil) to perform the micro-shear bond strength test, for which a chisel-shaped metal rod positioned at the interface of the resin cylinder and the tooth was used, and an incremental force was applied with a crosshead speed of 1 mm/min (20). Fracture toughness values were collected in Kgf and were converted to megapascals (MPa) using the following formula.

N (Newton) x mm2 = MPa

To determine the failure pattern, sample surfaces were examined under a trinocular stereomicroscope (AmScope SM20, United Scope LLC, USA) at 20x magnification. The samples were photographed and evaluated using image analysis software (AmScope).

-Statistical analysis 

SPSS v.23.0 for Windows (SPSS Inc., Chicago, Illinois) was used for data analysis. The normality of the data was previously evaluated with the Shapiro-Wilk test. The data did not have a normal distribution, so the Kruskal-Wallis test was used to evaluate the differences between groups. The Mann-Whitney U test was used to evaluate whether there were significant differences between the agents at 24 hours and one week. The significance level adopted was *p* < 0.05.

## Results

Table 2 shows the results of micro-shear bond strength 24 hours after cementation with RelyX U200 self-adhesive resin on dentin, the group that obtained the highest values of micro-shear bond strength was chlorhexidine (15.951 MPa); while the lowest values were observed in the AgNPs group (6.660 MPa). Significant differences were found between the AgNPs group and the other conditioning agents (*p* < 0.05). After seven days of storage, it was observed that the CHX 2% group obtained higher values (14.169 MPa) compared to the other groups; however, no significant differences were found (*p* > 0,05). When micro-shear bond strength was compared between the groups at 24 hours and seven days, it was found that there were no statistically significant differences between the groups (*p* > 0.05).

**Table 2:**
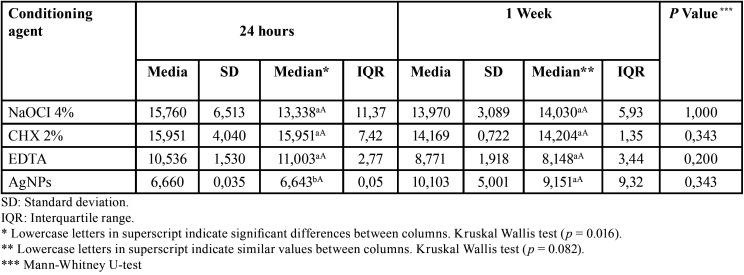
Micro-shear bond strength of RelyX U 200 self-adhesive resin on dentin at 24 hours and one week with surface pretreatment (MPa).

**Figure 1:**
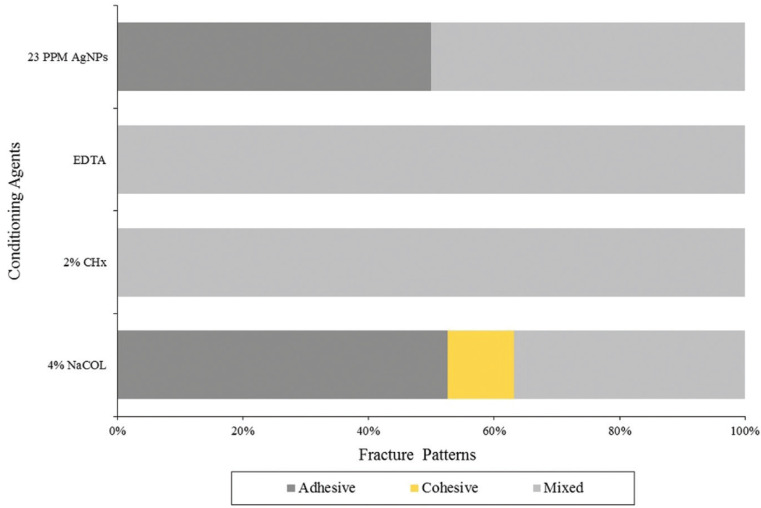
Distribution of failure patterns in the groups.

**Figure 2:**
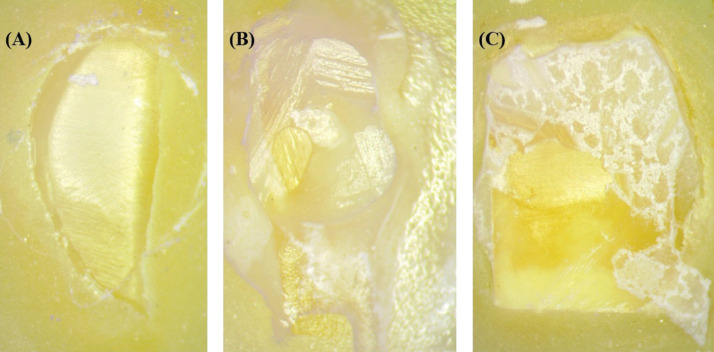
Representative samples show failure patterns. A. Adhesive, B. Cohesive, C. Mixed.

Figure 1 shows that a higher percentage of adhesive failures was observed in the silver nanoparticles (AgNPs) and sodium hypochlorite (NaOCI) groups. A higher percentage of mixed failures was recorded in the EDTA and chlorhexidine (CHX) groups, while the only group that recorded cohesive failure was NaOCI. Figure 2 shows the detail of the failure patterns.

## Discussion

The present study compared different conditioning agents applied on dentin prior to the bonding procedure with RelyX U200 self-adhesive resin. Micro-shear bond strength was also measured after 24 hours and one week to compare the differences between the groups.

Self-adhesive resin cements bond to tooth structures and restorative materials without requiring the application of a separate pretreatment or adhesive. They are easy to use and can be applied in one step. In addition, they release fluoride, tolerate moisture, and produce little or no postoperative sensitivity; however, they do not delete the smear layer (21). De Munck *et al*. showed that the acid component of RelyX Unicem has a weak demineralizing effect on dentin; even if applied to the fractured dentin surface without a smear layer, the demineralizing effect is not ideal. Their results showed that the bond strength between uncleaned dentin and RelyX Unicem is significantly lower than after NaOCI treatment, inconsistent with the manufacturer’s claims (22). Therefore, applying a pretreatment agent is indicated to improve the bonding ability of self-adhesive cements.

In this study, NaOCI was used as a pretreatment agent, shown to degrade proteins efficiently and may remove excess protein. Proteins interfere with establishing a clinically successful acid etch pattern, and their removal improves binding (23). CHX at 2% is one of the most investigated concentrations and shows the most significant inhibitory effect on MMPs and increased resistance to micro-cleavage. When CHX was used as an MMP inhibitor, its effect was measured immediately, and in the long term, it promoted different effects on the micro-shear bond strength of resin restorations. This fact was observed in the data obtained from a meta-analysis that showed differences in micro-shear bond strength between the groups evaluated immediately and at 24 h compared to the groups evaluated at six months, 12 months and 2-5 years, including aged or thermally cycled samples (24). EDTA is an MMP inhibitor solution capable of increasing the longevity of the adhesive interface by dissolving the mineral components of dentin without altering the stability of the organic matrix and without causing collagen denaturation. It maintains or even increases the average bond strength despite the adhesive system used (25). Silver nanoparticles play a crucial role in inhibiting bacterial growth in aqueous and solid media. The addition of AgNPs in water resulted in a homogeneous dispersion throughout the adhesive. This distribution improved the antibacterial ability of the composite despite the decrease in shear bond strength (26).

Kambara *et al*. compared the dentin bond strength of three self-adhesive cements with smear layer pretreatments using a calcium chelating agent (EDTA) and a deproteinizing solution (NaOCl). They found that for RelyX Unicem Clicker cement, pretreatment with EDTA did not affect dentin bond strength (8.6 MPa) (*p* > 0.05) while pretreatment with NaOCl for 15 s significantly increased bond strength (13.6 MPa). They concluded that pretreatments influenced the bond strength of self-adhesive cements (27). The results obtained in the present study showed higher values for both EDTA (10.536 MPa) and NaOCI (15.760 MPa), so their use likely promotes an increase in the bonding capacity of self-adhesive resins. de Oliveira *et al*. found that the application of AgNPs did not promote changes in bond strength values (6.66 MPa) (17), similar to those obtained in the present study (6.660 MPa), compared to the control group that did not apply any conditioning agent (9.76 MPa).

The adhesive failure type was predominant for the AgNPs group, reaching 50% of samples. This agrees with a study by Mohammed *et al*. (28) They observed that the failure mode was predominantly adhesive for the control group that obtained the lowest tensile strength values, with a higher percentage of mixed failures for the groups using disinfectants. They concluded that the higher percentage of mixed failures in disinfectant groups was attributed to increasing shear bond strength, which was reflected in the failure mode of the bonding system. The primary failure mode in samples with low adhesive strengths was an adhesive failure, whereas cohesive fractures in dentin or composite were observed with higher adhesive strength.

## Conclusions

It is concluded that the pretreatment agents influence positively the micro-shear bond strength of RelyX U200 self-adhesive cement applied on dentin, except for AgNPs, which, although they are used as antibacterial agents, do not present significant values about the shear bond strength of the self-adhesive resin cement.

## References

[1] Manso AP, Carvalho RM (2017). Dental Cements for Luting and Bonding Restorations: Self-Adhesive Resin Cements. Dent Clin North Am.

[2] Pedreira A, D'Alpino P, Pereira P, Chaves S, Wang L, Hilgert L (2016). Effects of the application techniques of self-adhesive resin cements on the interfacial integrity and bond strength of fiber posts to dentin. J Appl Oral.

[3] Weiser F, Behr M (2015). Self-adhesive resin cements: a clinical review. J Prosthodont.

[4] Pan Y, Xu X, Sun F, Meng X (2019). Surface morphology and mechanical properties of conventional and self-adhesive resin cements after aqueous ageing. J Appl Oral Sci.

[5] Mushashe AM, Gonzaga CC, Cunha LF, Furuse AY, Moro A, Correr GM (2016). Effect of Enamel and Dentin Surface Treatment on the Self-Adhesive Resin Cement Bond Strength. Braz Dent J.

[6] Shafiei F, Kamran S, Memarpour M, Aghaei T (2019). Bond strength and adhesive interfacial micromorphology of self-adhesive resin cements: Effect of reduced times of pre-etching. J Clin Exp Dent.

[7] Moghaddas MJ, Hossainipour Z, Majidinia S, Ojrati N (2017). Comparison of the shear bond strength of self-adhesive resin cements to enamel and dentin with a different application protocol. Electron Physician.

[8] Lin J, Shinya A, Gomi H, Shinya A (2010). Bonding of self-adhesive resin cements to enamel using different surface treatments: bond strength and etching pattern evaluations. Dent Mater J.

[9] Saleh NE, Guven MC, Yildirim G, Erol F (2019). Effect of different surface treatments and ceramic primers on shear bond strength of self-adhesive resin cement to zirconia ceramic. Niger J Clin Pract.

[10] Voegeli G, Bella ED, Mekki M, Machtou P, Bouillaguet S (2021). Effect of a Modified Irrigation Protocol on the Cleanliness of Moderately Curved Canals. Eur J Dent.

[11] Cecchin D, Farina AP, Galafassi D, Barbizam JV, Corona SA, Carlini-Júnior B (2010). Influence of sodium hypochlorite and EDTA on the microtensile bond strength of a self-etching adhesive system. J Appl Oral Sci.

[12] Silva AM, Alencar CM, Jassé FA, Pedrinha VF, Zaniboni JF, Dantas AA (2021). Using a self-adhesive cementation system, post-space irrigation with acid solutions on bond strength and dentin penetrability. J Clin Exp Dent.

[13] Breschi L, Cammelli F, Visintini E, Mazzoni A, Vita F, Carrilho M (2009). Influence of chlorhexidine concentration on the durability of etch-and-rinse dentin bonds: a 12-month in vitro study. J Adhes Dent.

[14] Bulut NB, Evlioğlu G, Röhlig BG, Çelakıl T (2018). Effect of dentin pretreatment on shear bond strength of three resin-based luting cements. Eur Oral Res.

[15] Tuncdemir AR, Yildirim C, Ozcan E, Polat S (2013). The effect of a diode laser and traditional irrigants on the bond strength of self-adhesive cement. J Adv Prosthodont.

[16] Faria-e-Silva AL, Menezes M de S, Silva FP, Reis GR, Moraes RR (2013). Intra-radicular dentin treatments and retention of fiber posts with self-adhesive resin cements. Braz Oral Res.

[17] de Oliveira Reis B, de Lima Godas AG, Suzuki TYU, Tozzi TCF, Briso ALF, Dos Santos PH (2020). Do Different Pretreatments of Dentine Surface Affect the Bond Strength with a Self-adhesive Resin Cement?. Oral Health Prev Dent.

[18] Jowkar Z, Shafiei F, Asadmanesh E, Koohpeima F (2019). Influence of silver nanoparticles on resin-dentin bond strength durability in a self-etch and an etch-and-rinse adhesive system. Restor Dent Endod.

[19] Gruber YL, Jitumori RT, Bakaus TE, Reis A, Gomes JC, Gomes GM (2020). Effect of applying different concentrations of EDTA on the adhesion of fiber posts using self-adhesive cements. Braz Oral Res.

[20] Latta MA, Tsujimoto A, Takamizawa T, Barkmeier WW (2020). Enamel and Dentin Bond Durability of Self-Adhesive Restorative Materials. J Adhes Dent.

[21] Gundogdu M, Aladag LI (2018). Effect of adhesive resin cements on bond strength of ceramic core materials to dentin. Niger J Clin Pract.

[22] De Munck J, Vargas M, Van Landuyt K, Hikita K, Lambrechts P, Van Meerbeek B (2004). Bonding of an auto-adhesive luting material to enamel and dentin. Dent Mater.

[23] Espinosa R, Valencia R, Uribe M, Ceja I, Saadia M (2008). Enamel. Enamel deproteinization and its effect on acid etching: an in vitro study. J Clin Pediatr Dent.

[24] Hamdan-Nassar T, Bellot-Arcís C, Paredes-Gallardo V, García-Sanz V, Pascual-Moscardó A, Almerich-Silla JM (2019). Effect of 2% Chlorhexidine Following Acid Etching on Microtensile Bond Strength of Resin Restorations: A Meta-Analysis. Medicine (Kaunas).

[25] Coelho A, Amaro I, Rascão B, Marcelino I, Paula A, Saraiva J (2020). Effect of Cavity Disinfectants on Dentin Bond Strength and Clinical Success of Composite Restorations-A Systematic Review of In Vitro, In Situ and Clinical Studies. Int J Mol Sci.

[26] Degrazia FW, Leitune VC, Garcia IM, Arthur RA, Samuel SM, Collares FM (2016). The effect of silver nanoparticles on an orthodontic adhesive's physicochemical and antimicrobial properties. J Appl Oral Sci.

[27] Kambara K, Nakajima M, Hosaka K, Takahashi M, Thanatvarakorn O, Ichinose S (2012). Effect of smear layer treatment on dentin bond self-adhesive cements. Dent Mater J.

[28] Mohammed HA, Ali GA, Baroudi K (2014). The effect of different disinfecting agents on bond strength of resin composites. Int J Dent.

